# Visit-to-Visit Variability and Seasonal Variation in Blood Pressure With Single-Pill Fixed-Dose Combinations of Angiotensin II Receptor Blocker/Calcium Channel Blocker and Angiotensin II Receptor Blocker/Diuretic in Hypertensive Patients

**DOI:** 10.14740/jocmr2292w

**Published:** 2015-08-23

**Authors:** Yuhei Shiga, Shin-ichiro Miura, Sen Adachi, Yasunori Suematsu, Makoto Sugihara, Atsushi Iwata, Eiji Yahiro, Hiroaki Nishikawa, Masahiro Ogawa, Keijiro Saku

**Affiliations:** a Department of Cardiology, Fukuoka University School of Medicine, Fukuoka, Japan; b Department of Molecular Cardiovascular Therapeutics, Fukuoka University School of Medicine, Fukuoka, Japan

**Keywords:** Visit-to-visit variability, Seasonal variation, Blood pressure

## Abstract

**Background:**

The visit-to-visit variability in blood pressure (BP) has been shown to be a strong predictor of cardiovascular events. It is not known whether anti-hypertensive therapy using a single-pill fixed-dose combination of angiotensin II receptor blocker (ARB)/calcium channel blocker (CCB) or ARB/diuretic (DI) in hypertensive patients affects the visit-to-visit variability and seasonal variation of BP.

**Methods:**

We enrolled 47 hypertensive patients who had received a single-pill fixed-dose combination of either ARB/CCB (n = 30) or ARB/DI (n = 17) for 15 months. Beginning 3 months after the start of ARB/CCB or ARB/DI treatment, we determined the visit-to-visit variability in BP expressed as the standard deviation (SD) of average BP and the seasonal variation in BP expressed as the SD of average BP in each season (spring, summer, fall and winter were defined as lasting from March to May, June to August, September to November and December to February, respectively) for a year.

**Results:**

There were no significant differences in baseline patient characteristics except for the prevalence of coronary artery disease and the percentage of CCB excluding amlodipine in the ARB/CCB group between the ARB/CCB and ARB/DI groups. There were no significant differences in the 1-year time course of systolic and diastolic BP (SBP and DBP) between the groups, although there were significant differences in SBP in August and November and DBP in December. Interestingly, the visit-to-visit variability and seasonal variation of BP in the ARB/CCB group were similar to those in the ARB/DI group.

**Conclusion:**

Single-pill fixed-dose combinations of ARB/CCB and ARB/DI had similar effects on visit-to-visit variability and seasonal variation in BP in hypertensive patients.

## Introduction

The visit-to-visit variability in blood pressure (BP) has been shown to be a strong predictor of cardiovascular disease (CVD), stroke and mortality independent of BP per se [1-5]. The 24-h means and standard deviations (SDs) (i.e., variabilities) of systolic BP (SBP), mean BP and diastolic BP (DBP) have been shown to be related to the rate and severity of target-organ damage [6]. In addition, seasonal differences in BP are important in the treatment of hypertensive patients. Generally, BP is lower in hot months than in cold months [7], and CVD mortality and morbidity peak in the winter.

Although optimal BP control is associated with remarkable clinical benefits with regard to CV and renal protection, many patients still show higher BP. Most patients with hypertension (HTN) require two or more drugs to achieve their target BP [8]. Various guidelines recommend the use of combinations of angiotensin II receptor blockers (ARBs) and calcium channel blockers (CCBs) or diuretics (DIs) [9, 10]. However, there is still some controversy regarding which single-pill fixed-dose combinations of ARB/CCB or ARB/DI are effective for the treatment of HTN.

Clinicians are often obliged to reduce the dosage of anti-hypertensive drugs, especially DIs, in summer to avoid excessive BP lowering. We hypothesized that a combination of ARB and CCB would have a more beneficial effect on the visit-to-visit variability and seasonal variation in BP than a combination of ARB and DI. Therefore, we investigated the visit-to-visit variability and seasonal variation in BP in patients with HTN who had received a single-pill fixed-dose combination of either ARB/CCB or ARB/DI.

## Methods

### Study patients

We enrolled 47 hypertensive patients who received a single-pill fixed-dose combination of either telmisartan 40 mg/day + amlodipine 5 mg/day (MicamloAP^®^) (n = 30, ARB/CCB group) or telmisartan 40 mg/day + hydrochlorothiazide 12.5 mg/day (MicombiAP^®^) (n = 17, ARB/DI group), and did not change these anti-hypertensive drugs for 15 months. The subjects were aged 20 years or older (no upper limit of age). The protocol in this study was approved by the ethics committee of Fukuoka University Hospital, and we retrospectively collected all of the data and performed a *post hoc* analysis using the database of Fukuoka University Hospital.

### Patient characteristics

The characteristics of the patients, with regard to history of dyslipidemia (DL), diabetes mellitus (DM), coronary artery disease (CAD) and medication use, were obtained from medical records. Patients with LDL-C ≥ 140 mg/dL, TG ≥ 150 mg/dL, and/or HDL-C < 40 mg/dL, or who were receiving lipid-lowering therapy, were considered to have DL. DM was defined using the Japanese Diabetes Society criteria or the use of a glucose-lowering drug. CAD (stable angina) was defined as no changes in the frequency, duration, or intensity of symptoms for 4 weeks and as lumen diameter stenosis > 50% by coronary angiography in at least one major coronary artery. Body mass index (BMI) was calculated as weight/height (kg/m^2^).

### Measurement of BP and pulse rate (PR)

BP was determined as the mean of two measurements obtained in an office setting by the conventional cuff method using a mercury sphygmomanometer after at least 5 min of rest. Office SBP, DBP and PR measurements were obtained every clinic visit at a 1- to 2-month interval for 15 months. Beginning 3 months after the start of ARB/CCB or ARB/DI treatment, we determined the visit-to-visit variability in BP or PR expressed as the SD of the average BP or PR and the seasonal variation in BP or PR expressed as the SD of the average BP or PR in each season for the next 12 months. Spring, summer, fall and winter were defined as lasting from March to May, June to August, September to November, and December to February, respectively, in Japan.

### Statistical analysis

Statistical analysis was performed using the Stat View statistical software package (Stat View 5; SAS Institute Inc., Cary, NC, USA). Data are shown as the mean ± SD. Categorical and continuous variables were compared between the groups by a Chi-square analysis and unpaired *t*-test, respectively. One-way analysis of variance was used to compare time course changes in BP and PR between the ARB/CCB and ARB/DI groups. A value of P < 0.05 was considered significant.

## Results

### Patient characteristics at baseline

Table 1 shows the characteristics in the ARB/CCB and ARB/DI groups. There were significant differences in %CAD and %CCB excluding amlodipine in the ARB/CCB group amlodipine between the ARB/CCB and ARB/DI groups. The %CAD in the ARB/DI group was significantly higher than that in the ARB/CCB group.

**Table 1:** Baseline Characteristics in the ARB/CCB and ARB/DI Groups

	ARB/CCB (n = 30)	ARB/DI (n = 17)
Age, years	70 ± 10	68 ± 10
Male, %	60	59
BMI, kg/m^2^	25 ± 3	24 ± 3
DM, %	13	6
DL, %	57	76
CAD, %	17	47*
Medications		
CCB, %	7	59*
DI, %	13	0
αβ blocker, %	7	12
β blocker, %	17	24
Aldosterone antagonist, %	7	12

Continuous variables are expressed as mean ± SD. ARB: angiotensin II receptor blocker; CCB: calcium channel blocker; DI: diuretic; BMI: body mass index; DM: diabetes mellitus; DL: dyslipidemia; CAD: coronary artery disease. %CCB in the ARB/CCB group and %DI in the ARB/DI group indicate the percentages of CCB except for amlodipine and DI except for hydrochlorothiazide, respectively. *P < 0.05 vs. ARB/CCB.

### Time courses of BP and PR in each month for 12 months

**Figure 1:**
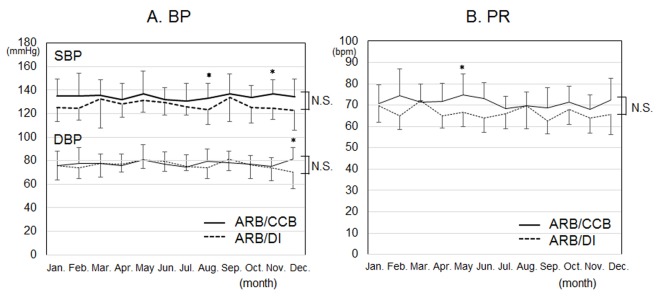
Time courses of blood pressure (BP) (A) and pulse rate (PR) (B) in each month for 12 months in the ARB/CCB and ARB/DI groups. *P < 0.05 vs. ARB/DI group. NS: not significant.

The time courses of BP and PR in each month for 12 months are shown in Figure 1. Although SBP in August and November, DBP in December and PR in May in the ARB/CCB group were significantly higher than those in the ARB/DI group, there were no differences in the 1-year time courses of BP and PR between the groups.

### Time courses of BP and PR in each season for 12 months

**Figure 2:**
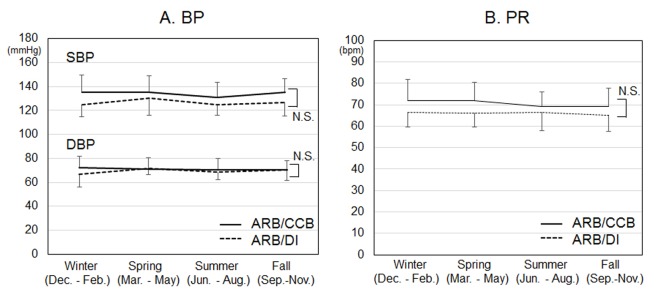
Time courses of systolic and diastolic blood pressure (SBP and DBP) (A) and pulse rate (PR) (B) in each season for 12 months. *P < 0.05 vs. ARB/DI group. NS: not significant.

The time courses of BP and PR in each season for 12 months are shown in Figure 2. There were no differences in the seasonal time courses of BP and PR in each season between the groups.

### Visit-to-visit variability and seasonal variation of BP and PR

**Figure 3:**
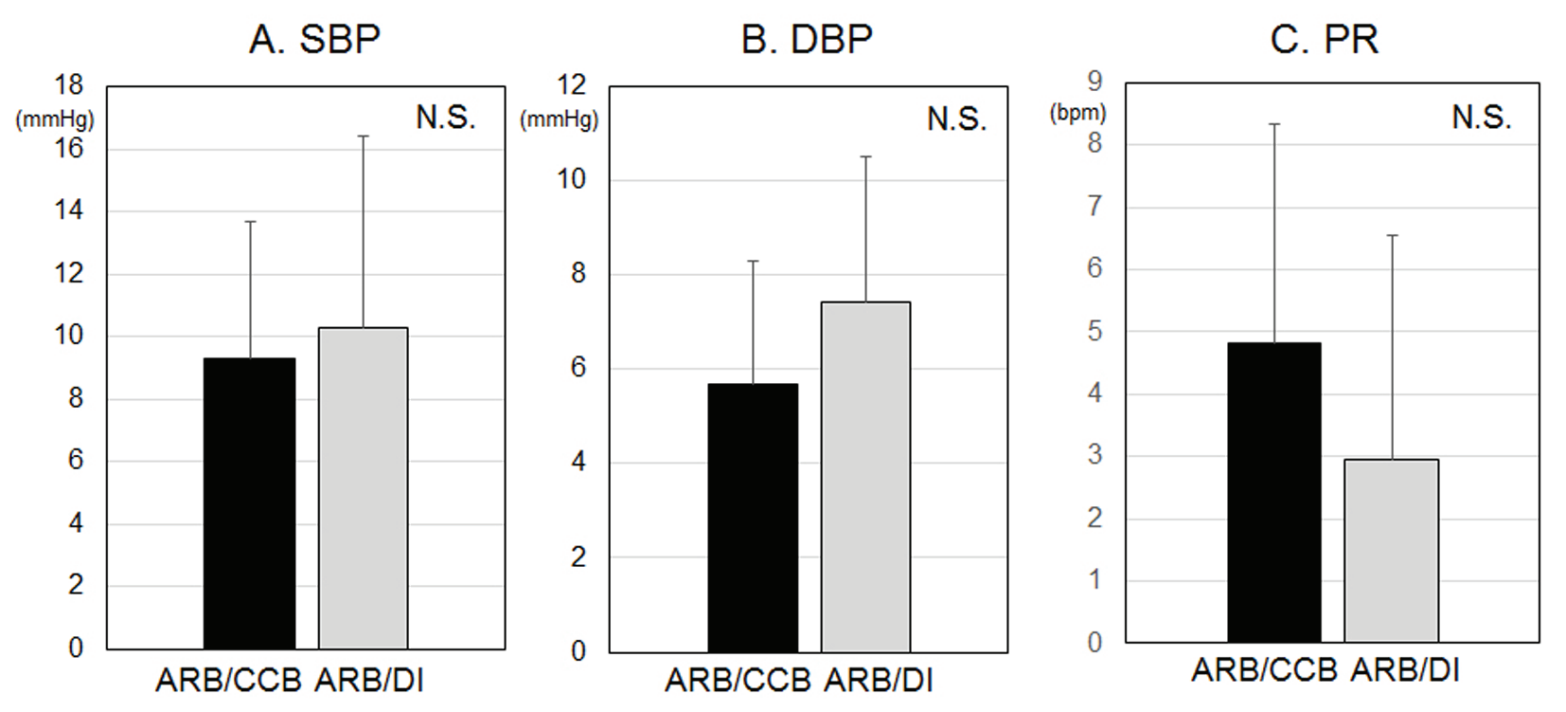
Visit-to-visit variabilities of systolic blood pressure (SBP) (A), diastolic BP (DBP) (B) and pulse rate (PR) (C). NS: not significant.

**Figure 4:**
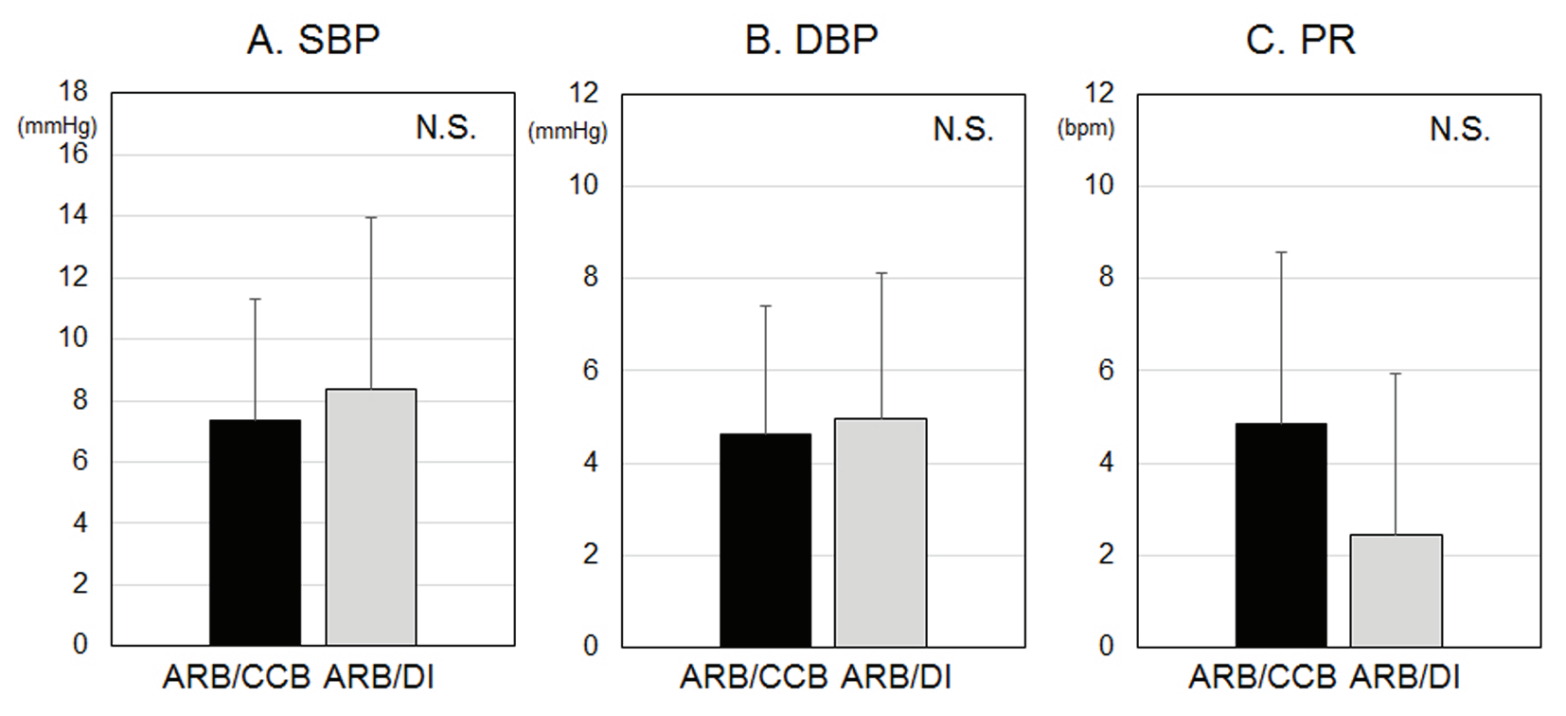
Seasonal variations of systolic blood pressure (SBP) (A), diastolic BP (DBP) (B) and pulse rate (PR) (C). NS: not significant.

There were no differences in the visit-to-visit variability of BP and PR, as shown in Figure 3. In addition, there were no differences in the seasonal variation of BP and PR, as shown in Figure 4.

## Discussion

In this retrospective study, we assessed the visit-to-visit variability and seasonal variation of BP and PR in patients with HTN who had received a single-pill fixed-dose combination of either ARB/CCB or ARB/DI. First, we found that single-pill fixed-dose combinations of ARB/CCB and ARB/DI similarly affected the visit-to-visit variability of BP and PR in hypertensive patients. Second, there was no difference in the seasonal variation between ARB/CCB and ARB/DI.

The most interesting finding was that ARB/CCB and ARB/DI similarly affected the visit-to-visit variability of BP. At the beginning of the study, we hypothesized that the combination of ARB/CCB would have a more beneficial effect on the visit-to-visit variability of BP than the combination of ARB/DI, since an increase in the visit-to-visit variability of BP may be explained in terms of arterial stiffness and abnormal autonomic function [11, 12]. In addition, CCB reduced the interindividual variability of SBP more than non-dihydropyridine CCB, non-loop DI, ARB, angiotensin converting enzyme inhibitor, α_1_-blocker and β-blocker [13]. Combination therapy with ARB/CCB may be preferable to that with ARB/DI for decreasing the visit-to-visit variability of BP [14]. There are several reasons why our results did not support our hypothesis. According to the Japanese Society of Hypertension Guidelines for the Management of Hypertension (JSH2014), the target in BP control should be < 140/90 mm Hg [9]. Since almost all of the patients in this study achieved the target BP, there were no differences in the visit-to-visit variability of BP. Moreover, a high visit-to-visit variability of BP is associated with cardiac diastolic function independent of mean BP [15]. Since all of the patients had HTN in this study and HTN first influences diastolic function, the differential effect in visit-to-visit variability of BP between the ARB/CCB and ARB/DI groups is not clearly seen. Moreover, since 59% of patients in the ARB/DI group were receiving CCB, it may also affect the visit-to-visit variability of BP.

Second, we thought that the combination of ARB/CCB would have a more beneficial effect on the seasonal variation of BP than the combination of ARB/DI because the dose of DI needs to be reduced in the summer to avoid excessive BP lowering due to dehydration. In addition, since a colder ambient temperature leads to higher BP [16] and high dietary salt intake potentiates the cold-induced increase in BP [17], DI may have a greater effect on the seasonal variation in BP than CCB. Interestingly, there were no differences in the seasonal variation of BP between the ARB/CCB and ARB/DI groups. The combination of ARB/DI could be used safely throughout the year.

There were no differences in the visit-to-visit variability or the seasonal variation of PR between the ARB/CCB and ARB/DI groups. Heart rate variability, a measure of autonomic dysfunction, has been associated with an increased risk of myocardial ischemia in patients with cardiovascular disease [18]. Although %CAD in the ARB/DI group was significantly higher than that in the ARB/CCB group, the combination of ARB/DI may be relatively safe in patients with CAD.

This study has several limitations. First, this study was retrospective and included a small number of patients. Second, the analysis was performed after other various anti-hypertensive treatments in addition to a combination of ARB/CCB or ARB/DI. Third, we measured BP and PR for only 1 year and did not evaluate the clinical outcome. Prospective long-term studies are needed to clarify these limitations.

In conclusion, single-pill fixed-dose combinations of ARB/CCB and ARB/DI had similar effects on the visit-to-visit variability and seasonal variation in BP in hypertensive patients.
